# Inhibitory effect of *Cnidium monnieri* aqueous extract on *Eimeria tenella* infection in chicks

**DOI:** 10.1016/j.psj.2025.106339

**Published:** 2025-12-26

**Authors:** Yanchun Wang, Haixia Han, Qiang Zhang, Zhe Zheng, Mohan Yang, Baihui Zhang, Yingjuan Lan, Tingting Yu, Yanan Cai

**Affiliations:** College of Veterinary Medicine, Jilin Agricultural University, Changchun 130118, China

**Keywords:** Eimeria tenella, Aqueous Extract of Cnidium monnieri, NF-κB

## Abstract

*Eimeria tenella* is the primary cause of cecal coccidiosis in broilers. This infection leads to severe intestinal inflammation and tissue damage, resulting in substantial economic losses to the poultry industry. The widespread use of chemical anticoccidials has led to serious drug resistance, thus creating an urgent need for the development of safe and natural alternatives. Cnidium monnieri (L.) Cusson, a traditional Chinese medicinal herb, possesses anti-inflammatory, antibacterial, and immunomodulatory properties. This study aimed to systematically evaluate the anticoccidial efficacy of the aqueous extract of *Cnidium monnieri* (CMAE) and to explore its mechanism of action both in vivo and in vitro.One hundred twenty one-day-old broilers were randomly divided into four groups: a negative control (CON), a positive control (PC or E. tenella infection), an E. tenella + CMAE group (*E. tenella* + CM), and an E. tenella + diclazuril group (*E. tenella* + DZ).

In vivo experiments showed that the E. tenella + CM group achieved an anticoccidial index (ACI) of 161.5, demonstrating a moderate anticoccidial effect comparable to that of the E. tenella + DZ group (ACI = 160). Histopathological analysis revealed that the cecal villus length in the E. tenella + CM group was 12.5 ± 0.8 cm at 10 days post-infection (dpi) (8.2 ± 0.5 cm in the *E. tenella* group, *P<0.01*). Additionally, the infiltration of inflammatory cells in cecal tissues was reduced by 62.3 ± 4.1% (*P<0.01*).

Serum enzyme-linked immunosorbent assay (ELISA) results demonstrated that, at 7 dpi, the serum levels of IgA (1.8 ± 0.2 μg/mL) and IgM (1.6 ± 0.1 μg/mL) in the E. tenella + CM group were significantly higher than the corresponding levels in the E. tenella group (*P<* 0.01).

In vivo quantitative real-time polymerase chain reaction (qPCR) results indicated that CMAE, at the tested concentration, significantly downregulated the mRNA expression of pro-inflammatory cytokines (IL-1β, IL-6, IL-17, IL-22, TNF-α) in infected cells (by 38.7%–52.1%) while simultaneously upregulating the mRNA expression of anti-inflammatory cytokines (IL-10, TGF-β) (by 1.8- to 2.1-fold; *P<* 0.01). Western blot analysis further confirmed that CMAE significantly inhibited the activation of the NF-κB pathway: the phosphorylation level of p65 was reduced by 51.2 ± 3.4%, the phosphorylation level of IκB-α was reduced by 46.8 ± 2.9%, and the expression of the anti-apoptotic protein Bcl-2 was decreased by 39.5 ± 2.7% (*P<* 0.01).

Flow cytometry analysis showed that, at 10 dpi, the peripheral blood CD4⁺/CD8⁺ T cell ratio in the E. tenella + CM group was 1.8 ± 0.1, which was significantly higher than that in the E. tenella group (*P<* 0.05) and comparable to the ratio in the CON group.

In conclusion, the aqueous extract of Cnidium monnieri effectively mitigated the intestinal damage induced by Eimeria tenella in broiler chickens by inhibiting the NF-κB signaling pathway, modulating cytokine balance, and enhancing both humoral and cellular immunity. These findings suggest its significant potential as a natural anticoccidial agent, thereby providing a scientific basis for the prevention and control of coccidiosis in broiler chickens.

## Introduction

Avian coccidiosis, an intestinal disease in broiler chickens caused by Eimeria protozoan parasites, results in annual global economic losses exceeding US 3 billion for the poultry industry (Zhang et al., 2024). China, due to its intensive farming model, accounts for approximately 15% of these total losses.Among the seven pathogenic Eimeria species infecting poultry, Eimeria tenella is considered the most detrimental due to its high host specificity and tissue tropism (Jiang et al., 2024). This parasite exclusively invades the cecal epithelial cells of broilers, where its schizogony-mediated destruction of the intestinal mucosal barrier triggers diffuse hemorrhagic enteritis, intestinal villus sloughing, and inflammatory cell infiltration.These pathological changes lead to retarded growth, reduced feed conversion efficiency, and, in acute infections, a mortality rate of up to 20% (Cai et al., 2022), collectively posing a major threat to the broiler farming industry.

Currently, avian coccidiosis is primarily controlled and prevented using chemical anticoccidial drugs and live vaccines (Arczewska-Włosek et al., 2023). However, the chronic overuse of chemical drugs (e.g., diclazuril, monensin) has resulted in severe drug resistance: drug-resistant E. tenella strains have been reported in over 80% of Chinese poultry farms, with the half-maximal effective concentration (EC₅₀) increasing 5.3-fold over the past decade, thus significantly diminishing the preventive and therapeutic efficacy of traditional drugs (Ewais et al., 2024). Simultaneously, drug residues in poultry meat and eggs also pose potential risks to human food safety (Emes et al., 2023). While live vaccines induce some immune protection, their cross-protection rate against heterologous strains is less than 40%, and they carry the risk of virulence reversion, which makes it challenging to manage coccidiosis control given the genetic diversity of Eimeria (Zheng et al., 2023). Consequently, the development of natural, low-toxicity, and multi-target alternative anticoccidial strategies is urgent (Chen et al., 2023).

Cnidium monnieri (L.) Cusson, the dried mature fruit of the Apiaceae family, is included in the Chinese Pharmacopoeia (Sun et al., 2020). In traditional Chinese medicine (TCM), it is commonly used to treat parasitic infections and dermal inflammation (Wang et al., 2024). Given the limitations of current control measures, *Cnidium monnieri* emerges as a promising candidate for developing a natural alternative. Its selection for this study is based on the rationale that a plant with documented anti-parasitic and immunomodulatory properties may circumvent the issues of drug resistance and residue associated with synthetics, while offering a multi-target approach potentially superior to the narrow spectrum of live vaccines. Modern phytochemical studies indicate that Cnidium monnieri contains over 350 bioactive components, with coumarins (e.g., osthole), flavonoids, and terpenoids being the core active substances that exhibit pharmacological effects such as anti-inflammatory, antifungal, and immunomodulatory activities (Lin et al., 2024). Prior studies have confirmed that Cnidium monnieri extracts can inhibit Eimeria oocyst sporulation in vitro and reduce parasite infectivity (Park et al., 2024).

However, a critical evaluation of the existing literature reveals a prominent gap. While these in vitro findings are promising, they lack validation in complex biological systems. There is a notable scarcity of comprehensive in vivo studies that systematically evaluate the anticoccidial efficacy, optimal dosing, safety profile, and underlying mechanisms of *Cnidium monnieri* extracts in avian hosts. This limitation hinders the translation of its potential from the laboratory bench to practical application in poultry farming. Therefore, conducting rigorous in vivo investigations is imperative to bridge this knowledge gap and assess its true therapeutic value against coccidiosis.

The aqueous extraction process, a classic method for isolating bioactive components from Chinese medicinal herbs, offers advantages such as operational simplicity, high safety, and good bioavailability (Su et al., 2024). The aqueous extract of Cnidium monnieri (CMAE) retains its water-soluble bioactive components (e.g., osthole glycosides). Furthermore, the intestinal microbiota can convert polymers within the extract into active aglycones, which further enhances its efficacy (Wang et al., 2024). Additionally, the natural supramolecular structures in CMAE can protect active components from gastric acid degradation and facilitate intestinal targeted release, which aligns well with the therapeutic requirements for intestinal diseases in broiler chickens (Li et al., 2019). Building on this rationale, the present study used an E. tenella-infected broiler chicken model and in vitro cell experiments to systematically explore, for the first time in a comprehensive in vivo setting, the anticoccidial effect and molecular mechanism of CMAE, with a focus on the NF-κB signaling pathway, aiming to provide an experimental and theoretical foundation for the development of natural anticoccidial agents.

## Materials and methods

### Experimental animals, parasites, and drugs

• Experimental Animals: A total of 120 healthy 1-day-old white-feathered broilers were purchased from the Changchun Academy of Agricultural Sciences. The chickens were housed in sterile wire cages under controlled conditions: an ambient temperature of 28–32°C, 50–60% relative humidity, and a 16 h light: 8 h dark (16L:8D) cycle. They were fed with antibiotic-free feed and had *ad libitum* access to drinking water.
• Parasites: Eimeria tenella oocysts were kindly provided by the Parasitology Laboratory at Jilin Agricultural University. The oocysts were preserved in 2% potassium dichromate solution at 4°C and sporulated in a constant-temperature incubator at 28°C for 48 h before use (Sun et al., 2024).
• Drugs: Dried Cnidium monnieri fruits (voucher specimen no. CM202403) were purchased from the Changchun Fubai Cao Pharmacy and authenticated by Professor Li Yalan (Jilin Agricultural University) based on morphological and phytochemical characteristics. Diclazuril (purity ≥ 98%) was purchased from Ningxia Terui Pharmaceutical Co., Ltd. (Yinchuan, China).

A total of 120 one-day-old broilers were randomly allocated into four groups with 30 chickens per group: the uninfected control (CON), the *Eimeria tenella*-infected group (PC), the *E. tenella* + CMAE treatment group (CM), and the *E. tenella* + diclazuril treatment group (DZ). For molecular and histopathological analyses, tissue and blood samples were obtained via terminal sampling. At each time point (3, 5, 7, and 10 days post-infection), 5 chickens were randomly selected from each group and euthanized for sample collection (n = 5 per group per time point). The remaining chickens in each group (25 chickens) were maintained for daily monitoring of body weight, survival, and fecal oocyst output, which are required for calculating the Anticoccidial Index (ACI). The sample size (n=5) for terminal sampling was determined to be adequate based on preliminary data and power considerations. Throughout the study, chickens were housed with 5 cages per treatment group (6 birds per cage), serving as biological replicates. All molecular assays were performed with three technical replicates.

### Main reagents and instruments

The main reagents used were: Madin-Darby bovine kidney (MDBK) cells (ATCC, USA); DMEM high-glucose medium (Gibco, USA); fetal bovine serum (FBS, Gibco, USA); phosphate-buffered saline (PBS, Solarbio, China); CCK-8 reagent (Beyotime Biotechnology, China); RIPA lysis buffer (Solarbio, China); protease and phosphatase inhibitor cocktails (Biosharp, China); TRIzol reagent (Invitrogen, USA); PrimeScript™ RT Kit (Takara, Japan); TB Green® Premix Ex Taq™ II (for qPCR, Takara, Japan); BCA protein quantification kit (EpiZyme, China); and Omni-ECL™ chemiluminescence detection kit (EpiZyme, China). Rabbit monoclonal antibodies targeting β-actin (Servicebio, China), Bcl-2 (Affinity, USA), p65 (Affinity, USA), p-p65 (Affinity, USA), I-κB (Affinity, USA), and p-I-κB (Affinity, USA), HRP-conjugated goat anti-rabbit IgG secondary antibody (SouthernBiotech, USA); chicken IgM and IgA ELISA kits (Shanghai Preferred Bioscience, China); and the CD3-FITC, CD4-PE, and CD8-APC fluorescent antibodies (BD Biosciences, USA). Hematoxylin-Eosin (HE) staining reagents (Solarbio, China); paraffin (Leica, Germany); 2.5% potassium dichromate solution (Sinopharm, China); and a chicken peripheral blood lymphocyte separation kit (Solarbio, China). Dried Cnidium monnieri fruits (voucher specimen no. CM202403); diclazuril (purity ≥ 98%, Ningxia Terui Pharmaceutical, China), ethanol at gradient concentrations (Sinopharm Chemical Reagent Co., Ltd., China); and 4% paraformaldehyde (Solarbio, China).

Main instruments were: ultraclean workbench; cell culture incubator (37°C, 5% CO₂), microplate reader; ABI 7500 Real-Time Quantitative PCR System; inverted microscope with an integrated imaging system; Western blot apparatus; and a chemiluminescence imager. Paraffin microtome; automated paraffin embedding system; and a Nikon Eclipse E100 microscope.Electronic analytical balance; flow cytometer; and software including ImageJ, GraphPad Prism (v 9.5.1), and SPSS (v 26.0).

### Preparation of the aqueous extract of Cnidium monnieri (CMAE)

Fifty grams of dried *Cnidium monnieri* fruits were pulverized and soaked in 500 mL of distilled water for 4 hours. The mixture was boiled at 100°C (high heat) for 15 minutes and then simmered (low heat) for 1 hour. The filtrate was collected using 8 layers of sterile gauze, and the residue was re-extracted with 300 mL of distilled water. The combined filtrates were concentrated under reduced pressure (60°C, 0.08 MPa) to a concentration of 1 g/mL (1 g of crude drug per mL) and sterilized through a 0.22 μm filter membrane. CMAE was stored at 4°C.

### Experimental design

The uninfected group served as the negative control (CON group), and the infected group served as the positive control (*E. tenella* group). Two additional treatment groups were included: the E. tenella + Cnidium monnieri group (*E. tenella* + CM), and the E. tenella + diclazuril group (*E. tenella* + DZ). All four groups were provided with the same feed and water ad libitum for the first 12 days. On day 13, treatments commenced. The CMAE dose (0.2 mL/chicken, 0.2 g crude drug/mL) was determined via preliminary dose-gradient experiments: low (0.1 mL/chicken, ACI = 132.7, no significant oocyst inhibition), medium (0.2 mL/chicken, optimal efficacy + good safety), and high (0.3 mL/chicken, no improved ACI [P > 0.05] but mild intestinal discomfort). The diclazuril dose (0.5 mg/kg body weight, 1 mL premix/chicken) follows clinical recommendations in the *Veterinary Drug Use Guidelines*.

The *E. tenella* + CM group received 0.2 mL CMAE daily via oral gavage; the *E. tenella* + DZ group received diclazuril (0.2 mL/L drinking water, fresh daily); the CON group received 0.2 mL PBS as a vehicle control. All treatments were administered once daily for 10 consecutive days.

Except for the CON group, the other groups were orally inoculated with 1 × 10⁴ sporulated *Eimeria tenella* oocysts on day 14 to establish the infection model.

The in vivo anticoccidial efficacy of the Cnidium monnieri aqueous extract was assessed by calculating the Anticoccidial Index (ACI) for each group. (mean weight gain in *E. tenella* group ÷ mean weight gain in uninfected CON group) × 100%. The survival rate was calculated as: number of surviving chicks ÷ total number of chicks × 100%. Oocyst quantification was performed using the McMaster method. Fecal samples (2 g each) were collected daily from days 4 to 8 post-infection (D4 to D8 PI), homogenized with an appropriate volume of saturated saline, filtered, and the oocyst count was performed using a McMaster chamber. The average oocyst count per day was recorded.

The ACI was calculated as follows:

ACI=(relativeweightgainrate+survivalrate)−(cecaopisthotonosumindex+grosslesionscore.)

An ACI ≥ 180 is considered excellent, 160 ≤ ACI *<* 180 is considered good, 120 ≤ ACI *<* 160 is considered moderate, and ACI *<* 120 is considered ineffective (Kim et al., 2024).

### Quantitative PCR (qPCR) detection of cytokine mRNA expression

Total RNA was extracted from cecal tissue samples. The tissues were first homogenized by grinding with liquid nitrogen and then transferred to a centrifuge tube containing 1 mL of total RNA extraction reagent (Takara, Japan). Complementary DNA (cDNA) was synthesized using the All in One First Strand cDNA Synthesis SuperMix (All in One, Beijing, China) for subsequent quantitative real-time PCR (qPCR).All primers were synthesized by Bioengineering Ltd. (listed in Table 1). Relative mRNA expression was detected by qPCR using the All-Style Gold® Tip Green qPCR SuperMix (All-Style Gold, Beijing, China).All reactions were performed in triplicate (Table 1).

**Table 1:** Primer sequences for quantitative real-time PCR analysis.

Gene name	Primer name	Sequence	Reference sequence
β-actin	β-actinF β-actinR	5′-TGTTACCAACACCCACACCC-3′ 5′-TCCTGAGTCAAGCGCCAAAA-3′	NM_205518.1
IL-10	IL-10F IL-10R	5′-TCACTTCCTCCTCCTCATCA-3′ 5′-GAGACGTTCGAGAAGATGGATG-3′	NC_052557.1
IL-17	IL-17F IL-17R	5′-CTTCTGAGGCATTTGGAAGC-3′ 5′-ACTGGGCGGTCATAGAACAG-3′	NM204460.1
IL-22	IL-22F IL-22R	5′-GGTTGTCTTCTGCTGTTGTTGCTG-3′ 5′-GCCAAGGTGTAGGTGCGATTCC-3′	NM_001199614.1
IL-6	IL-6-F IL-6-R	5′-CAAGGTGACGGAGGAGGAC-3′ 5′-TGGCGAGGAGGGATTTCT-3′	AJ309540
TNF-α	TNF-α-F TNF-α-R	5′-CGGGACGGATGAGAAGAAC-3′ 5′-TCGGCGCTCCAGATGTAC-3′	NM31160.1
TGF-β	TGF-β-F TGF-β-R	5′-CATCGAGCTCTTCCAGATCC-3′ 5′-GACATCGAAGGACAGCCACT-3′	NM 205454.1

### Western blotting (WB) analysis

Cecal tissue samples were homogenized by grinding in liquid nitrogen and then transferred to a centrifuge tube containing 1 mL of RIPA lysis buffer (Solarbio, Beijing, China). Subsequently,12 μL of both protease and phosphatase inhibitor cocktails (Biosharp, Anhui, China) were added.The tissue was incubated at 4°C for 30 minutes before being centrifuged at 13680 × g for 5 minutes to extract total proteins. Protein concentration was determined using the BCA Protein Quantification Kit (Epizyme Biotech, Shanghai, China). Samples were separated by SDS-PAGE and transferred to a PVDF membrane. The membrane was blocked with 5% skimmed milk powder at room temperature for two hours. Following blocking, the membrane was incubated overnight at 4°C with antibodies against p65 (Affinity, BF8176), p-p65 (Affinity, 87AF3391), Bcl-2 (Affinity, AF6139), IκBα (Affinity, AF5002), p-IκBα (Affinity, AF3239), and β-actin (Servicebio, 88GB15003). The next day, after washing three times with TBST, the membrane was incubated with the appropriate HRP-conjugated secondary antibodies for 2h at room temperature.

### Histopathological examination

Cecal tissue samples were collected and fixed in 4% paraformaldehyde solution. The fixed tissues were then paraffin-embedded and sectioned. ly, histopathological sections were prepared and stained using the Hematoxylin and Eosin (HE) method.

### Serum ELISA assay

The peripheral blood of chickens was collected in a 1.5 mL centrifuge tube and left to stand at 37°C for 30 minutes, then kept at 4°C overnight. The resulting serum was then aspirated and stored at -80°C. The serum concentrations of IgM and IgA were measured using the Chicken Immunoglobulin M (IgM) ELISA Kit and Chicken Immunoglobulin A (IgA) ELISA Kit from Shanghai Preferred Bioscience and Technology Co.

### Blood flow Cytometry

Fresh blood 2mL was collected from the four chicken groups on post-feeding days 7 and 10 into EDTA anticoagulant tubes, mixed thoroughly, and placed on a shaker to prevent coagulation. An equal volume of PBS was added to each tube for dilution, and lymphocytes were separated using the Chicken Peripheral Blood Lymphocyte Isolation Solution Kit (Solarbio, Beijing, China). After cell separation, the cells were counted and diluted, then centrifuged at 380 × g for 5 minutes at 4°C.Following centrifugation, 100 μL of supernatant was removed, the remaining cells were re-suspended, and 5 μL of chicken serum was added to each tube for a 15-minute incubation. Subsequently, pre-diluted antibodies against CD3^+^, CD4^+^, and CD8^+^ were added to new centrifuge tubes, and single-antibody controls for CD3^+^, CD4^+^, and CD8^+^ were simultaneously prepared. The tubes were incubated for 35 minutes at 4°C, protected from light. Following antibody incubation, 1 mL of pre-diluted PBS buffer containing 2% serum was added to each tube and centrifuged at 380 × g for 5 minutes at 4°C. Then, 200 μL of supplementary solution was added, mixed well, and the cell suspension was filtered through a 200-mesh nylon mesh soaked in 75% alcohol and illuminated with UV light overnight. The filtered cells were transferred to flow cytometry tubes and kept on ice until analysis.

### Statistical analysis

All experiments were performed in triplicate (n=3). Experimental data of each group were processed using Image J and GraphPad Prism 9.5.1. Two-way ANOVA and one-way ANOVA were applied to analyze the differences among the experimental data of each group. *: P<0.05 indicates significant difference; **: P<0.01, ***: P<0.001, and ****: P<0.0001 all indicate extremely significant differences; ns: P>0.05 indicates no significant difference.

## Results

### Anti-coccidial effect of aqueous extracts of Cnidium monnieri

**Fig. 1:**
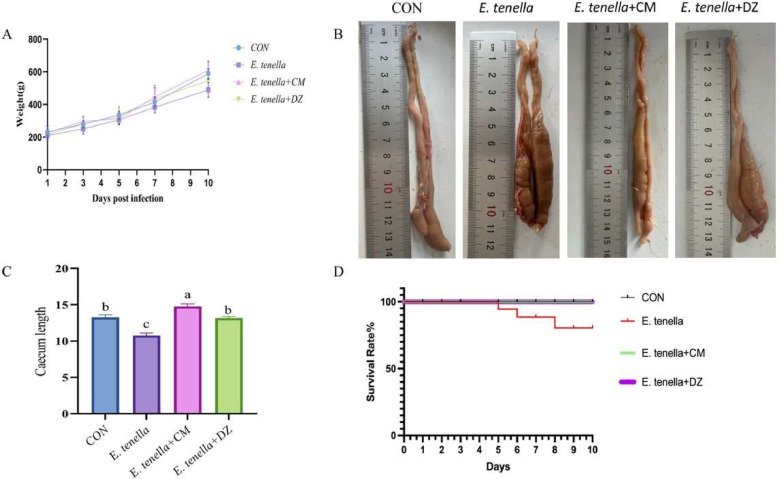
Effects of Cnidium monnieri aqueous extract on growth performance in Eimeria tenella-infected broilers (A) Changes in body weight of broilers in each group during 10 days post-infection. The E. tenella+CM group exhibited rapid body weight gain after the fifth day, reaching a final weight slightly higher than the CON group. (B) Macroscopic appearance of ceca showing hemorrhagic swelling and shortening in the E. tenella group, with significant recovery in both treatment groups. (C) Comparison of cecal length among groups at 10 dpi. The E. tenella+CM group showed significantly restored cecal length (12.5 ± 0.8 cm) compared to the E. tenella group (8.2 ± 0.5 cm, *P<* 0.01). (D) Survival rates of broilers in each group following E. tenella challenge, with the E. tenella group showing 80% survival and treatment groups maintaining 100% survival.

As shown in Fig. 1A, which illustrates the changes in body weight of chickens in each group ten days after the infection, the body weight of each group increased gently from days 1 to 5 post-infection. The growth trend was similar to the *E. tenella*+CM group, which exhibited rapid body weight gain after the fifth day. In contrast, the *E. tenella* group consistently showed slower body weight gain compared to the other three groups. By day 10, the *E. tenella*+CM group was slightly heavier than the CON group.

The results of the anticoccidial index are shown in Table 2. The *E. tenella*+CM and *E. tenella*+DZ groups achieved moderate anticoccidial effects with indexes of 161.5 and 160, respectively. The *E. tenella* group exhibited the worst anticoccidial effect, with an index of 56.7.

**Table 2:** Determination results of anti coccidiosis index in each group of chickens.

Group	Survival Rate(%)	Relative Weight Gain Rate(%)	Lesion Value	Oocyst Value	ACI
*CON*	100	100	0	0	200
*E. tenella*	70	58	31.3	40	56.7
*E.tenella+CM*	100	94	12.5	20	161.5
*E.tenella+DZ*	100	90	20	10	160

Additionally, as shown in Fig. 1B, the ceca comparison revealed that the *E. tenella* group had a shortened ceca with hemorrhagic swelling of the intestinal wall. In contrast, the *E. tenella*+CM and *E. tenella*+DZ groups showed recovery of the ceca and significant improvement in ceca shortening. Fig. 1C shows that the cecal length in the *E. tenella* + CM group (marked as “a”) was significantly longer than that in the *E. tenella* group (marked as “c”, *P* *<* *0.05*) and the CON and *E. tenella* + DZ groups (both marked as “b”, *P* *<* *0.05*). The cecal length in the *E. tenella* group was significantly shorter than the other three groups (*P* *<* *0.05*), while no significant difference was observed between the CON group and *E. tenella* + DZ group (P > 0.05). Fig. 1D shows that after *E. tenella infection*, mortality in the *E. tenella* group started from the fifth day, and by the tenth day the survival rate was 80%. Meanwhile, both the *E. tenella* + CM and *E. tenella* + DZ groups maintained a 100% survival rate, which was comparable to the CON group. These clinical observations confirm that CMAE effectively alleviates *E. tenella*-induced cecal structural damage (shortening and hemorrhagic swelling) and improves the survival rate of infected broilers, which is a key basis for its anticoccidial efficacy.

### Results of H and E staining of pathological sections of the ceca

**Fig. 2:**
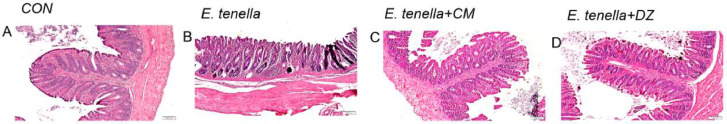
Histopathological evaluation of cecal tissues by HE staining (100 ×) (A) Normal cecal villi architecture with intact epithelial structure in uninfected control group. (B) Severe pathological damage in E. tenella-infected group, characterized by extensive villus shedding, shortened crypts, and massive inflammatory cell infiltration. (C) Remarkable restoration of villus structure and significantly reduced inflammatory cell infiltration (62.3 ± 4.1% reduction, *P<* 0.01) in CMAE-treated group. (D) Improved intestinal morphology with reduced inflammation in diclazuril-treated positive control group.

As shown in Fig. 2. The HE staining results of ceca tissue showed that the *E. tenella* group had severe ceca villi shedding, shortened ceca villi, severe damage to the intestinal villi, massive inflammatory cell infiltration, shortened crypts, and significantly higher damage than the other three groups. Compared with the *E. tenella* group the *E. tenella*+CM group and the *E. tenella*+DZ group showed growth of ceca villi, reduced inflammatory cell infiltration, and reduced intestinal villi shedding. Hematoxylin-eosin (HE) staining further confirms that the *E. tenella* group exhibited extensive cecal villus shedding, shortened crypts, and massive inflammatory cell infiltration, while the *E. tenella* + CM group had intact villus structures, with a 62.3 ± 4.1% reduction in the number of inflammatory cells compared to the *E. tenella* group (*P* *<* *0.01*). This indicates that CMAE inhibits excessive inflammatory response in the cecum, which is crucial for protecting intestinal mucosal integrity.

### Effect of Cnidium monnieri on antibody levels in broiler chickens infected with E. tenella

**Fig. 3:**
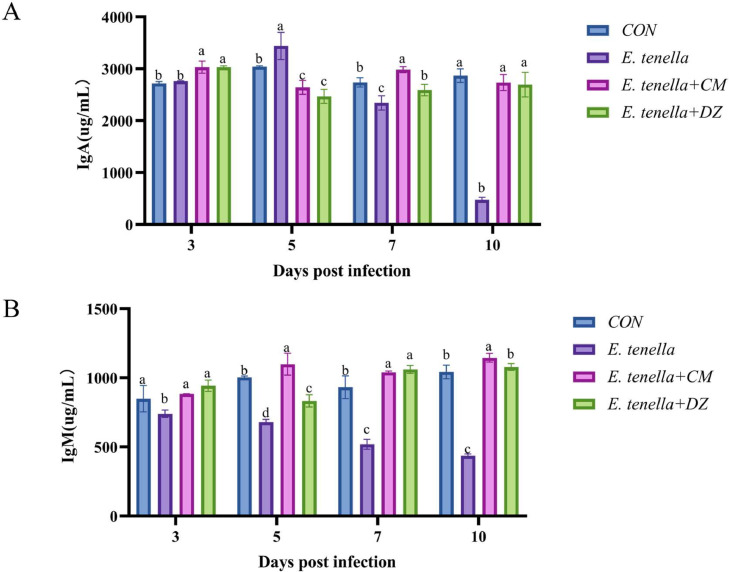
Effects of CMAE on humoral immunity in Eimeria tenella-infected broilers Serum levels of immunoglobulin A (IgA) and immunoglobulin M (IgM) measured by ELISA at multiple time points post-infection. E. tenella infection significantly reduced antibody levels, with IgA beginning to decrease from 5 dpi and IgM from 3 dpi. CMAE treatment significantly restored both IgA (1.8 ± 0.2 μg/mL) and IgM (1.6 ± 0.1 μg/mL) levels at 7 dpi compared to the infected group (*P<* 0.01), demonstrating enhanced humoral immune response.

The *E. tenella* infection significantly reduced the serum levels of IgA (Fig. 3A) and IgM (Fig. 3B) in chickens compared with the CON group (*P* *<* *0.05*): the IgA levels in the *E. tenella* group gradually decreased from 5 dpi (marked as "c" vs. "a" in CON group), while IgM levels began to decline from 3 dpi (marked as "b" vs. "a" in CON group). In contrast, CMAE treatment (*E. tenella*+CM group) significantly restored both antibody levels: at 7 dpi, IgA levels (1.8 ± 0.2 μg/mL, marked as "a") and IgM levels (1.6 ± 0.1 μg/mL, marked as "a") in the *E. tenella*+CM group were comparable to the CON group (P > 0.05) and significantly higher than those in the *E. tenella* group (*P* *<* *0.01*). The *E. tenella*+DZ group (positive control) also showed a similar antibody recovery trend, confirming that CMAE enhanced the humoral immune response in infected chickens. Notably, the restoration of IgA levels synergizes with the improved intestinal barrier structure (observed in HE staining) to form a dual defense: IgA prevents parasite adhesion to intestinal epithelial cells, while the intact intestinal mucosa blocks parasitic invasion—an integrated protective effect that strengthens the host’s resistance to *E. tenella* reinfection.

### Effect of aqueous extracts of Cnidium monnieri inflammatory factors in ceca tissues

**Fig. 4:**
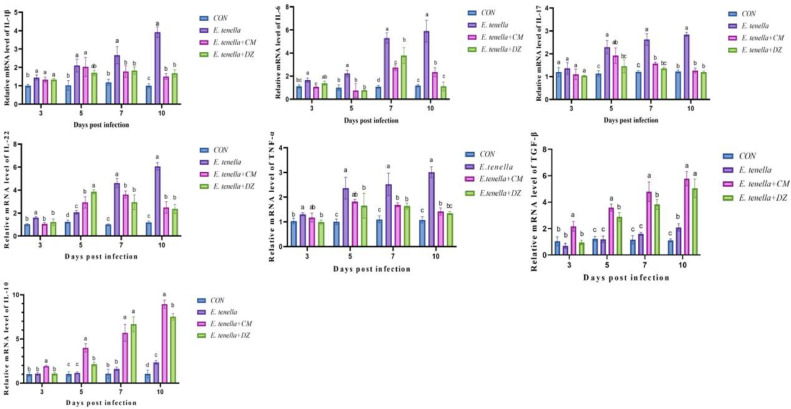
Modulation of cytokine mRNA expression in cecal tissues by CMAE Relative mRNA expression levels of pro-inflammatory cytokines (IL-1β, IL-6, IL-17, IL-22, TNF-α) and anti-inflammatory cytokines (TGF-β, IL-10) in cecal tissues. CMAE treatment significantly downregulated pro-inflammatory cytokine expression (reduced by 38.7%-52.1%) while simultaneously upregulating anti-inflammatory cytokine production (increased by 1.8-2.1 fold) at 10 dpi compared to the E. tenella group (*P<* 0.01). Notably, CMAE showed superior promotion of anti-inflammatory cytokine expression compared to diclazuril during 7-10 dpi.

Effect of aqueous extracts of *Cnidium monnieri* on inflammatory factors in cecal tissues. Inflammatory factors are key mediators of immune regulation post-infection. In the *E. tenella* group, the mRNA expression levels of pro-inflammatory cytokines (*TNF-α, IL-17, IL-6, IL-1β, IL-22*) in cecal tissues remained comparable to the CON group within 3 dpi, but gradually elevated from 3 dpi onward (marked as “a” or “b” vs. CON group “c”, *P* *<* *0.05*). In contrast, anti-inflammatory cytokine (IL-10, *TGF-β*) expression in the *E. tenella* group was persistently suppressed across all time points (*P* *<* *0.05* vs. CON group). After CMAE treatment (*E. tenella*+CM group): Pro-inflammatory cytokines: From 5 dpi, the mRNA expression of *TNF-α, IL-17, IL-6, IL-1β*, and *IL-22* was significantly downregulated by 38.7%–52.1% (marked as “c” vs. *E. tenella* group “a”, *P* *<* *0.01*), and restored to levels comparable to the CON group (P > 0.05) by 10 dpi; Anti-inflammatory cytokines: The expression of IL-10 and *TGF-β* was significantly upregulated from 3 dpi onward (1.8–2.1-fold increase vs. *E. tenella* group, *P* *<* *0.01*), with levels even higher than the CON group at 7–10 dpi (marked as “a” vs. CON group “b”, *P* *<* *0.05*). Notably, between 7–10 dpi, CMAE showed a superior ability to promote anti-inflammatory cytokine (IL-10, *TGF-β*) expression compared to the diclazuril group (*E. tenella*+DZ), whose anti-inflammatory factor levels were only restored to CON group levels (P > 0.05 vs. CON group) (Fig. 4).

### Effect of aqueous extracts of Cnidium monnieri on ceca tissue-associated proteins

**Fig. 5:**
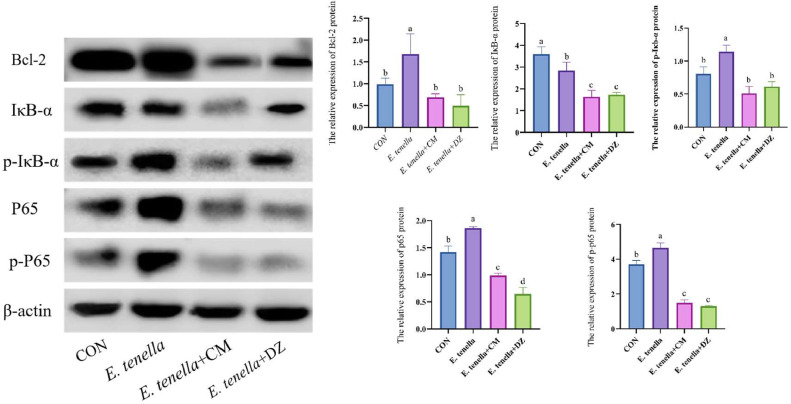
Effects of CMAE on NF-κB pathway activation and apoptosis-related proteins in cecal tissues Western blot analysis demonstrating CMAE's inhibition of NF-κB signaling pathway. Infection with E. tenella significantly increased phosphorylation of p65 and IκBα proteins and upregulation of anti-apoptotic protein Bcl-2. CMAE treatment effectively suppressed this activation, reducing p65 phosphorylation by 51.2 ± 3.4%, IκBα phosphorylation by 46.8 ± 2.9%, and Bcl-2 expression by 39.5 ± 2.7% compared to the infected group.

As shown in Fig. 5 this protein is recognised by Bcl-2, IκB-α, p-IκBα, p65, p-p65 antibodies. It was found that infection with *E. tenella* increased the levels of phosphorylated proteins P65 and I-κBα and the production of apoptotic protein Bcl-2.

### Peripheral blood flow cytometry

**Fig. 6:**
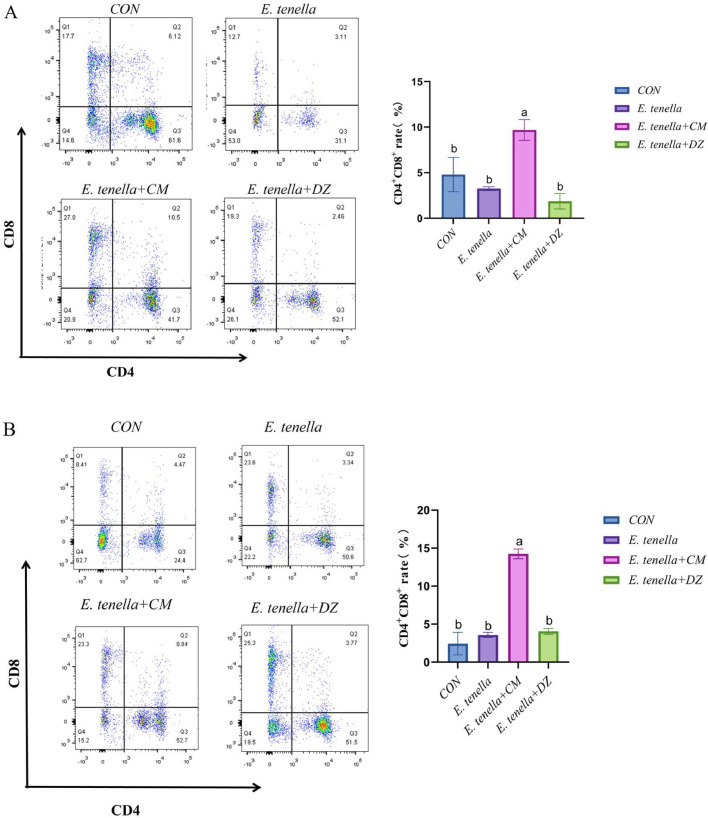
Impact of CMAE on T lymphocyte subpopulations in peripheral blood Flow cytometric analysis of CD4⁺/CD8⁺ T cell ratios in peripheral blood lymphocytes at (A) 7 days and (B) 10 days post-infection. CMAE treatment significantly improved the CD4⁺/CD8⁺ T cell ratio, reaching 1.8 ± 0.1 at 10 dpi, which was significantly higher than the E. tenella group (*P<* 0.05) and comparable to the CON group. This restoration indicates enhanced cellular immune function and reestablished immune homeostasis.

After infection with *Eimeria tenella* coccidia in chickens, the effect of the drug on T cell activation in the broiler intestine was evaluated by flow cytometry to detect the expression of CD4^+^ and CD8^+^ in lymphocytes CD3^+^ in the peripheral blood on the 7th and 10th day in each group. The results, as shown in Fig. 6, showed that after treatment with the aqueous extract of *Cnidium monnieri*, the percentage of lymphocyte CD4^+^ and CD8^+^ double positivity in the peripheral blood on the 7th and 10th days were significantly increased compared with the other three groups (*P* *<* *0.05*). Concurrently, the normalized CD4⁺/CD8⁺ T cell ratio indicates a restoration of cellular immune homeostasis: CD4⁺ T cells regulate inflammatory responses by modulating cytokine secretion, while CD8⁺ T cells directly kill parasite-infected cells. This balanced ratio is consistent with the improved cytokine profile observed earlier, collectively underscoring the comprehensive immunomodulatory capacity of CMAE.

## Discussion

Cecal coccidiosis caused by *Eimeria tenella* remains a major threat to the global broiler industry. It disrupts the integrity of cecal epithelial cells, triggers intestinal mucosal damage and inflammatory cell infiltration, leading to growth retardation and increased mortality in broilers, and ultimately results in substantial economic losses. This is corroborated by Liu et al. (2024), who demonstrated that *Eimeria tenella*-like intestinal inflammation directly impairs key production parameters (e.g., weight gain and feed efficiency) in broilers, providing a mechanistic explanation for the associated economic impact (Liu et al., 2024). Currently, the prevention and control of this disease rely heavily on chemical anticoccidial drugs (e.g., diclazuril) and live vaccines. However, drug resistance of *Eimeria tenella* has become a widespread challenge in Chinese poultry farms. This is corroborated by a systematic epidemiological survey (Sun et al., 2023), which reported that over 80% of farms in Zhejiang Province harbored resistant strains. In that study, resistance was comprehensively evaluated in vivo using four indices (ACI, POAA, RLS, ROP), revealing a detailed resistance profile for the Yiwu isolate (e.g., severe resistance to salinomycin and nicarbazine). Additionally, live vaccines exhibit poor cross-protection against heterologous strains (less than 40%) with the risk of virulence reversion. Therefore, there is an urgent need for natural, low-toxicity alternative solutions, and traditional Chinese herbal medicines have emerged as research hotspots due to their multi-targeted effects and low tendency to induce drug resistance.

*Cnidium monnieri* (L.) Cusson, a traditional Chinese medicinal herb recorded in the Chinese Pharmacopoeia, contains more than 350 bioactive components, among which coumarins (e.g., osthole, bergapten, imperatorin) and flavonoids (e.g., cnidimoside A) are the core active substances (Xu et al., 2024). Specifically, Xu et al. (2024) demonstrated that osthole—the most well-characterized coumarin component—exerts anti-inflammatory effects by suppressing the activation of the NF-κB signaling pathway (via reducing IκB degradation and p65 phosphorylation) and downregulating the expression of pro-inflammatory cytokines (e.g., *IL-6*) in lipopolysaccharide (LPS)-stimulated inflammatory models. Additionally, aqueous extracts of *C. monnieri* can inhibit the sporulation of *Eimeria* oocysts, including the clinically prevalent *E. tenella, E. acervulina*, and *E. necatrix* (Liao et al., 2024), with this inhibitory effect being more pronounced in mixed-species infection models (e.g., *E. acervulina-E. tenella* co-infection) that are dominant in commercial broiler flocks. In this study, in vivo broiler models and in vitro MDBK cell models were used to systematically evaluate the anticoccidial efficacy of *Cnidium monnieri* aqueous extract (CMAE) and explore its underlying mechanisms, providing a basis for research on herbal anticoccidial agents.

The anticoccidial index (ACI) is a core indicator for evaluating the efficacy of anticoccidial agents, integrating relative weight gain, survival rate, lesion severity, and oocyst excretion (Fries-Craft et al., 2023).

The anticoccidial index (ACI) of the *E. tenella* + CMAE group reached 161.5, which was comparable to that of the conventional synthetic anticoccidial drug diclazuril (ACI = 160) and fell within the effective range of ACI values reported for well-documented plant-derived anticoccidials (e.g., matrine, artemisinin derivatives). More importantly, as a multi-component herbal extract, CMAE is less likely to induce drug resistance compared to single-target synthetic drugs, which addresses the key limitation of current chemical control strategies and provides a promising natural alternative for sustainable coccidiosis management.

These findings align with the anticoccidial effects of other herbal extracts such as Sophora flavescens and Garcinia kola (Jiang et al., 2024), but CMAE’s unique advantage lies in its synchronous restoration of intestinal structural integrity and immune function—an effect rarely emphasized in previous herbal anticoccidial studies. CMAE’s dual regulation not only alleviates acute tissue damage but also enhances long-term intestinal immunity, which is critical for preventing reinfection in commercial broiler flocks.

Cytokine imbalance is a key factor in intestinal inflammation induced by *Eimeria tenella* infection. Pro-inflammatory cytokines (*IL-1β, IL-6, IL-17, IL-22, TNF-α*) exacerbate inflammation, while anti-inflammatory cytokines (IL-10, *TGF-β*) maintain intestinal homeostasis (Yang et al., 2023). Our qPCR analysis revealed that CMAE treatment effectively reversed this imbalance by significantly downregulating key pro-inflammatory cytokines and upregulating anti-inflammatory mediators. Notably, CMAE showed a more potent effect in promoting anti-inflammatory cytokine expression than diclazuril in the late stage of infection (7–10 dpi), reflecting a more sustained immunomodulatory effect.

This cytokine-regulating effect of CMAE is closely associated with the balance between T helper 17 (Th17) cells and regulatory T (Treg) cells (Cheng et al., 2024). The downregulation of Th17-related cytokines (*IL-17, IL-22*) and upregulation of Treg-related cytokines (IL-10, *TGF-β*) in the *E. tenella* + CM group suggest that CMAE can restore the Th17/Treg balance. This mechanism is consistent with that of osthole (a core component of *Cnidium monnieri*), which has been shown to inhibit Th17 cell differentiation and promote Treg cell proliferation in autoimmune disease models (Liu et al., 2020). This “immune balance reconstruction” mechanism distinguishes CMAE from chemical drugs that exert non-specific anti-inflammatory effects, as it mitigates pathological inflammation without disrupting the host’s normal parasitic clearance capacity—representing a more precise therapeutic strategy.

The nuclear factor κB (NF-κB) signaling pathway is a core pathway mediating intestinal inflammation induced by *Eimeria tenella*. After infection, IκBα is phosphorylated and degraded, releasing the p65 subunit to translocate into the nucleus, which then drives the transcription of pro-inflammatory cytokines and anti-apoptotic genes (e.g., Bcl-2) (Shan et al., 2023). Our Western blot analysis confirmed that CMAE treatment significantly inhibited the activation of this pathway, as evidenced by reduced phosphorylation of key proteins (p65 and IκBα) and downregulation of Bcl-2. This is consistent with previous studies showing that *Cnidium monnieri* extracts inhibit NF-κB activation in inflammatory bowel disease (Mukherjee et al., 2024), and our study further extends this finding by confirming the NF-κB pathway as a key target of CMAE in anti-*Eimeria tenella* infection—establishing a direct link between the herb’s traditional use and modern molecular mechanisms.

Humoral immunity (mediated by IgA and IgM) and cellular immunity (dominated by T lymphocytes) are important lines of defense for the host to clear *Eimeria tenella* and prevent reinfection (Li et al., 2021). Our immunological assays demonstrated that CMAE treatment effectively restored both humoral and cellular immune parameters in infected chickens. This comprehensive immunomodulation highlights CMAE’s potential to address the limitations of current strategies that focus solely on antiparasitic or anti-inflammatory effects. Compared with diclazuril, which only inhibits parasite replication without regulating immune function, CMAE’s ability to enhance both humoral and cellular immunity provides a more sustainable solution for coccidiosis control, especially in drug-resistant strain prevalence areas. Notably, the restoration of IgA levels synergizes with the improved intestinal barrier structure to form a dual defense (Wang et al., 2024), which is an integrated protective effect rarely reported for single-component anticoccidials.

## Conclusion

This study confirms that *Cnidium monnieri* aqueous extract exerts anti-*Eimeria tenella* effects through multiple dimensions: first, it protects intestinal pathological structure by reducing villus damage and restoring cecal integrity; second, it regulates cytokine balance to maintain intestinal inflammatory homeostasis by modulating the Th17/Treg axis; third, it inhibits the NF-κB signaling pathway to reduce pro-inflammatory gene transcription and anti-apoptotic protein expression; fourth, it enhances humoral and cellular immunity by increasing antibody levels and optimizing T cell immune function. Additionally, its aqueous extraction process (boiling and simmering of fruits) retains water-soluble active components without toxic solvent residues, and its natural supramolecular structure enables targeted release in the intestine, providing both safety and practicality. In summary, *Cnidium monnieri* aqueous extract shows significant potential as a natural alternative to chemical anticoccidial drugs. Future research should focus on isolating key active components and optimizing extraction processes to improve its efficacy, thereby contributing to the sustainable prevention and control of broiler coccidiosis.

## Funding

Key Research and Development Project of Jilin Provincial Department of Science and Technology,

Authorization number 20220202062NC.

## CRediT authorship contribution statement

**Yanchun Wang:** Writing – review & editing, Validation, Supervision, Software, Formal analysis, Data curation. **Haixia Han:** Writing – review & editing, Writing – original draft, Validation, Software, Methodology, Investigation, Formal analysis, Data curation. **Qiang Zhang:** Writing – original draft, Validation, Software, Data curation. **Zhe Zheng:** Supervision, Software, Methodology, Investigation. **Mohan Yang:** Supervision, Software, Methodology, Investigation. **Baihui Zhang:** Supervision, Methodology, Investigation. **Yingjuan Lan:** Supervision, Software, Investigation. **Tingting Yu:** Supervision, Investigation. **Yanan Cai:** Supervision, Resources, Project administration, Methodology, Funding acquisition.

## Disclosures

The authors declare no conflicts of interest.
